# Utility of Routine Post Kidney Transplant Anti-HLA Antibody Screening

**DOI:** 10.1016/j.ekir.2024.02.1394

**Published:** 2024-02-18

**Authors:** Sofiane Salhi, Nicolas Congy-Jolivet, Anne-Laure Hebral, Laure Esposito, Guillaume Vieu, Jean Milhès, Nassim Kamar, Arnaud Del Bello

**Affiliations:** 1 Department of Nephrology and Organ Transplantation, CHU Rangueil, Toulouse, France; 2 Faculté de santé, Université Paul Sabatier, Toulouse, France; 3 Molecular Immunogenetics Laboratory, EA 3034, Faculté de Médecine Purpan, IFR150 (INSERM), France; 4 Department of Immunology, CHU de Toulouse, Hôpital de Rangueil, CHU de Toulouse, France; 5 Etablissement Francais du Sang, CHU de Purpan, Toulouse, France; 6 INSERM U1043, IFR–BMT, CHU Purpan, Toulouse, France; 7 INSERM U1297, IFR–BMT, CHU Rangueil, Toulouse, France

**Keywords:** donor-specific antibodies, screening strategy, stratified medicine

## Abstract

**Introduction:**

*De novo* donor-specific antibody (dnDSA) is a strong biomarker associated with the development of antibody-mediated rejection (AMR) and graft loss after kidney transplantation. This procedure is expensive; however, systematic annual screening was recommended by some national organ transplant agencies or societies even though its clinical utility was not clearly established.

**Methods:**

To address this question, we retrospectively assessed the incidence of dnDSA according to the test justification (clinically indicated or systematic) in a cohort of low-immunological risk patients, defined by being nonhuman leukocyte antigen (non-HLA)-sensitized and having no previous kidney transplants.

**Results:**

A total of 1072 patients, for whom 4611 anti-HLA tests were performed, were included in the study. During the follow-up period of 8 (interquartile range, IQR: 5–11) years, 77 recipients developed dnDSA (prevalence of 7.2%). Thirty-five of these dnDSAs (45.5%) were detected during the first year posttransplantation. In 95% of patients with dnDSA, an immunizing event was identified in their medical records. dnDSA was detected in 46 of 4267 systematic screening tests (1.08%) performed. Active and chronic AMR were frequently observed in biopsies performed after systematic DSA testing (17.9% and 15.4%, respectively).

**Conclusion:**

Our results suggest that the detection by systematic screening of dnDSA in low-immunological risk kidney transplant patients without sensitizing events is a rare event, especially after 1 year. Moreover, in real life, systematic annual screening for dnDSA, seems having a limited impact to detect AMR at an earlier stage compared to patients in whom dnDSA was detected after a clinically indicated test.

## Graphical abstract

**Figure:**
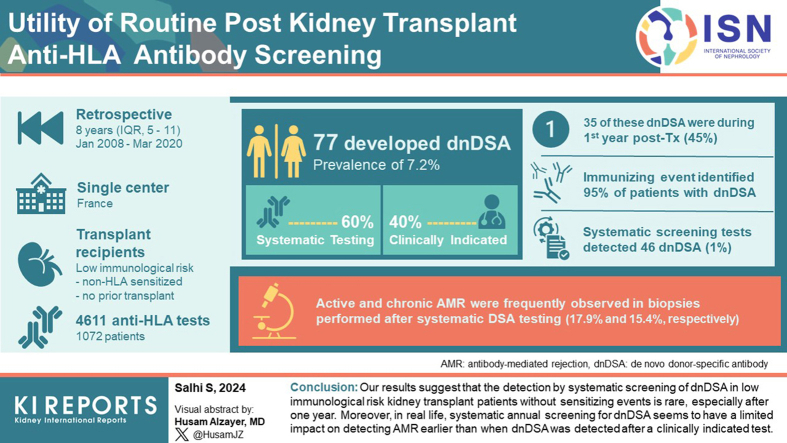

Kidney transplant survival remains lower than overall patient survival.^1^ AMR is currently the leading cause of graft failure in patients who return to dialysis.^2^^,^^3^ Recent advances in alloimmune risk stratification before transplantation, mainly driven by highly sensitive detection of preformed anti-HLA DSAs with the Luminex single antigen technology, have allowed the identification of the highest risk scenarios for rapid loss of function.^4^^,^^5^ After transplantation, the development of dnDSA has been strongly associated with the development of acute and chronic AMR and graft loss.^6^, ^7^, ^8^ However, the prevention of dnDSA development after transplantation remains a major obstacle. The effectiveness of current maintenance immunosuppressive regimens used to prevent dnDSA occurrence^9^ is counterbalanced by their side effects (e.g., infection, cancer, and cardiovascular disease development)^10^ that have, over time, prompted clinicians to seek lower doses of these treatments. Moreover, nonadherence is a major contributor to dnDSA development.^7^^,^^9^ To date, available treatments developed to tackle acute and chronic AMR remain disappointing.^11^

A screening strategy is expected to align with the widely accepted World Health Organization principles.^12^ The decision to implement disease screening generally considers the ability of a test to detect the disease at an early stage, the availability of effective treatment, and improved prognosis through early detection.^12^ In this context, whereas some groups have proposed testing patients for dnDSA when suitable situations occur (i.e., posttransplant immunizing event, immunosuppression minimization, and suspicion of nonadherence), systematic annual screening was recommended by some experts^13^ and organ transplant agencies.^14^^,^^15^ However, the clinical utility of this recommendation is not supported by the literature.

In the present study, we retrospectively assessed in a real life situation, the annual rate of dnDSA detected by systematic screening in a cohort of patients considered to be at low immunological risk (first kidney transplant, non-HLA sensitized). We also compared histological findings in patients with dnDSA detected during systematic screening with those in whom dnDSA was detected after a clinically indicated test.

## Methods

The study was reported according to the STROBE guidelines.^16^

### Study Population

Between January 2008 and March 2020, 2502 kidney transplantations were performed in our institution. All kidney transplant recipients with no detectable anti-HLA antibodies at transplantation who received a first transplant and who were screened for dnDSA were included in the study. Non-HLA-sensitized patients who had undergone a second kidney transplantation (*n* = 368), those transplanted previously with a nonrenal organ (*n* = 24), those who received combined transplantation (liver and kidney, *n* = 35; pancreas and kidney, *n* = 123), and HLA-sensitized patients (historical or at transplantation, *n* = 732) were excluded. Patients who presented with graft failure before 3 months and who were not screened for anti-HLA DSA after transplantation (*n* = 106) were also excluded, as well as transplant patients tapering immunosuppressants in order to start dialysis (*n* = 32). Thus, 1072 kidney transplant recipients were included in the study and followed-up with until graft loss or the end of follow-up period on June 30, 2022 (Supplementary Figure S1).

According to French law (Loi Jardé), anonymous retrospective studies do not require institutional review board approval. The clinical and research activities being reported are consistent with the Principles of the Declaration of Istanbul as outlined in the Declaration of Istanbul on Organ Trafficking and Transplant Tourism.

### Immunological Analyses

Systematic anti-HLA DSA screening was performed after transplantation at month 3 and 12 posttransplantation and annually thereafter, as recommended by the French Agency of Biomedicine.^17^

From January 2008 to June 2018, the presence of dnDSA was investigated using the Labscreen single antigen technology (One Lambda, Canoga Park, CA). The Labscreen single antigen determined the specificity of class I HLAs in A/B/Cw and class II in DR/DQ/DP IgG antibodies in the recipients’ sera according to the manufacturer’s instructions. The presence and specificity of antibodies were then detected using a Labscan 100, and the mean fluorescence (baseline value) for each sample in each bead was evaluated. A baseline mean fluorescence intensity value of >1000 was considered positive. Thereafter, from July 2018, to the end of the follow-up period (June 30, 2022), the presence of dnDSA was detected using the Lifecodes single antigen technology (LMX deluxe Immucor, Gateway Drive, GA). The Lifecodes single antigen (LSA class I/II) determined the specificity of class I HLAs in A/B/Cw and class II in DR/DQ/DP IgG antibodies in the recipients’ sera according to the manufacturer’s instructions. The presence and specificity of antibodies were then detected and the mean fluorescence intensity for each sample in each bead was evaluated. A mean fluorescence intensity value of >1000 was considered positive.

The donor HLA Cw and DP typing was not systematically available. DSA detection in these loci was confirmed by HLA typing a posteriori in case of posttransplant *de novo* anti-HLA antibody detection. All anti-HLA screening tests were checked and confirmed by our local immunologist (NC).

The immunodominant DSA was the DSA with the higher mean fluorescence intensity at detection. The mean fluorescent intensity sum was the sum of all A/B/Cw/DR/DQ/DP mean fluorescent intensity of the DSAs. To investigate the donor and recipient compatibility at the molecular level (i.e., to distinct the epitopes-related molecular configurations that can be recognized by the recipient HLA antibodies), HLA eplets mismatches were assessed after imputation from low resolution to high resolution with the HLAr package using the HLA matchmaker algorithm (ABC Eplets matching v4.0, and DRDQDP Eplets matching v 3.1, www.epitopes.net).

### Definition of “Clinically Indicated” or “Systematic Screening”

In our department, a written justification is required to perform anti-HLA screening after transplantation. Based on the patient’s electronic medical records, the prescription of a screening test for anti-HLA antibodies was considered to be “clinically indicated” (“performed for cause”) if the physician specifically mentioned a reason other than “systematic screening.” Moreover, to reduce biases related to prescription mistake, all justifications were reviewed by 2 nephrologists (SS and ADB) according to the biology recorded previously and at DSA screening.

### Histological Analyses

All histological findings were reviewed and scored by a renal transplant pathologist according to the Banff 2019 classification.^18^ In order to standardize conclusion diagnoses we reassessed all the biopsies attached diagnoses with the Banff-automation tool.^19^ We also assessed for each biopsy using the semisupervised clustering approach RejectClass tool.^20^^,^^21^

### Statistical Analyses

Statistical analyses were performed using R (v.4.4.2), HLAr packages (class I and II epitope load, alleilic mismatches), and survminer packages. Reported values represent the means (±SD) or medians (IQR). Quantitative variables were compared using the *t*-test or Mann-Whitney nonparametric test if appropriate. Categorical variables are expressed as percentages and compared between groups using the chi-square tests or, if appropriate, Fisher exact test. A *P*-value of <0.05 was considered statistically significant. To assess predictive factors for dnDSA development, all statistically significant values in univariate analysis were entered in several Cox-regression multivariable models, if the threshold of 10 events per variable was reached (follow-up from transplantation to June 30, 2022 or to the date of dnDSA detection). We built different models, including the variables (model 1: “HLA A, B, DR, DQ allele mismatches,” “recipient age,” “donor age;” model 2: “HLA class I mismatches,” “recipient age,” “donor age;” model 3: “HLA class II mismatches,” “recipient age,” “donor age;” or model 4 “HLA A, B, DR, DQ eplets mismatches,” “recipient age,” “donor age”). Proportional hazard assumption was checked by the graphic method (Supplementary Table S1). Because the dataset was complete concerning the variable included in the models, no imputation strategy was required. The final model reported in the study was the fourth model, based on the lowest Akaike Information Criteria score (AIC: 877).

## Results

### Incidence of dnDSA During Follow-Up

**Figure 1:**
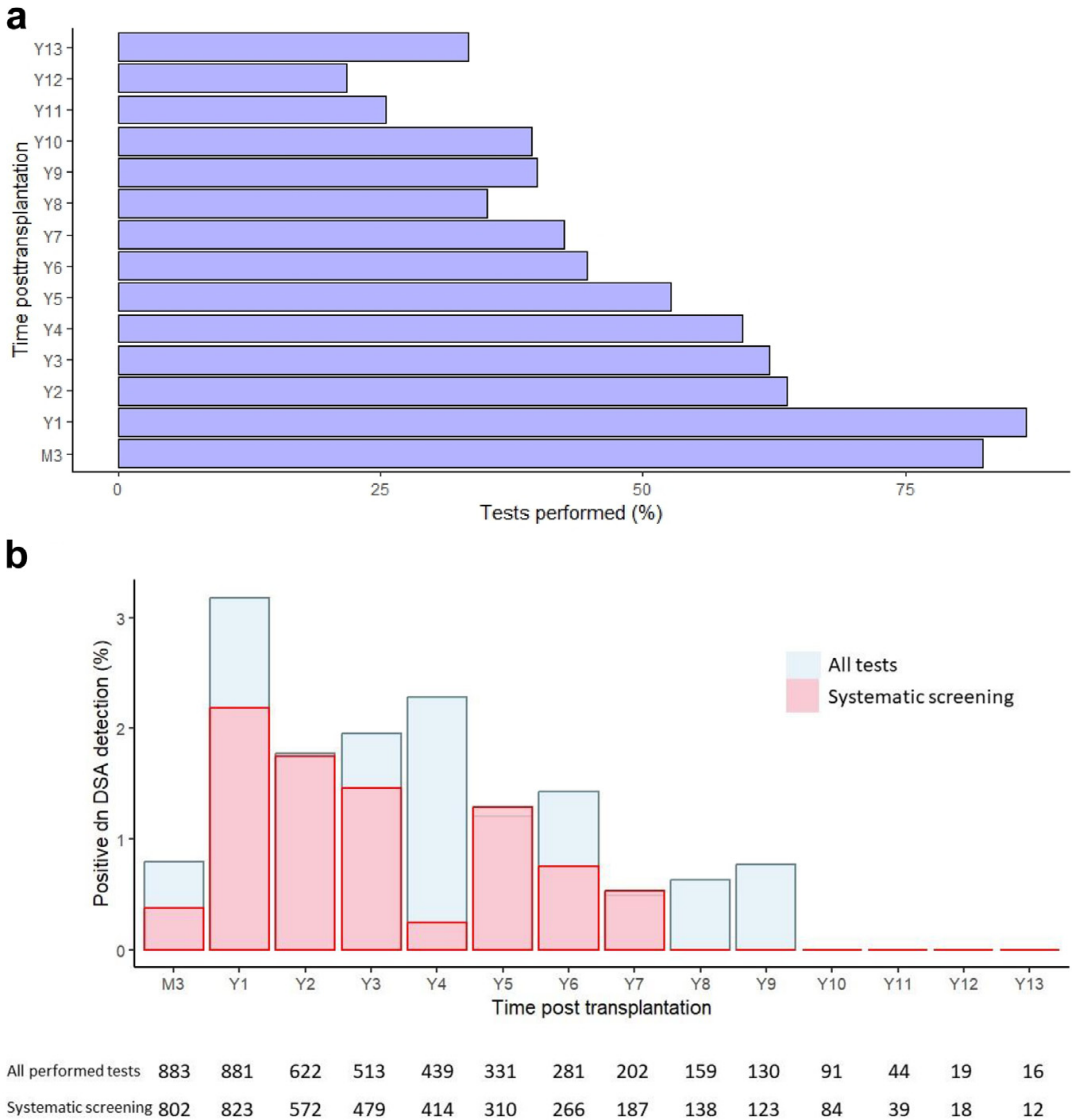
(a) Percentage of performed or theoretical tests during follow-up. The number of possible tests at each point was determined during the follow-up period by the functional status of the transplant at the mentioned time. The number of possible tests at each point was 1072, 1019, 976, 827, 740, 629, 629, 475, 326, 231, 172, 87, and 48 respectively at month 3, and years 1, 2, 3, 4, 5, 6, 7, 8, 9, 10, 11, 12, and 13. (b) Rate of detection of *de novo* DSA in all (blue) and annual systematic screening, during follow-up for all patients. Results are expressed as the percentage of positive tests among all performed tests. dnDSA, *de novo* donor specific antibodies; M3, month 3; Y, year.

A total of 1072 nonsensitized kidney transplant recipients were included; and 4611 tests were performed during the follow-up period. The median number of anti-HLA screenings per patient was 4 (IQR: 3–6), during a median follow-up of 8 (IQR: 5–11) years. The rate of tests effectively performed at each time posttransplantation varied from 86.4% at year 1 to 21.8% at year 12 (Figure 1a). Seventy-seven patients (7.2%) were identified as having dnDSA. dnDSA was detected in 60% of patients using systematic screening and in 40% of patients in whom tests for dnDSA were clinically indicated. Twelve patients developed anticlass I (15.6%), 53 patients developed anticlass II (68.8%), and 12 patients (15.6%) developed both class I and II dnDSA. The main characteristics of patients, regardless of developing DSA, is presented in Table 1. The proportion of patients who received an induction was similar in both dnDSA-positive and dnDSA-negative recipients (573 [57.6%]) in DSA-negative vs. 58 [75.3%] in dnDSA positive, *P* = 0.34). A large majority of patients received a triple initial, tacrolimus-based therapy in both groups (971 [97.6%] vs. 75 [97.4%], p=0.45), associated with mycophenolic acid (927 (93.2%) vs 73 (94.8%), p=0.45), and steroids (987 (99.2%) vs 77 (100%), *P* = 0.45). The proportion of patients who received a calcineurin inhibitor-based therapy remained similar at last follow-up (Table 1). Nine of the 77 patients (11.7%) who developed dnDSA presented with a history of biopsy-proven T cell-mediated rejection before dnDSA detection. This was higher than the rate of T cell-mediated rejection diagnosed in patients who did not develop dnDSA (2.5%, *P* = 0.0004). Previous studies have suggested the importance of molecular mismatches in the development of dnDSA.^9^^,^^22^ Both the anti-A/B (14.0 ± 6.9 in DSA negative vs. 16.0 ± 6.1 in patients who developed dnDSA, *P* < 0.0001), and DR/DQB1 epitope load (6.0 ± 6.0 vs. 19.0 ± 11.0, *P* < 0.0001) were significantly higher in patients who developed dnDSA during the follow-up. After multivariate analysis, a higher recipient age (adjusted hazard ratio = 0.97, 95% confidence interval [0.95–0.99], *P* = 0.01) was the sole protective factor for DSA formation, whereas a higher sum of HLA class I and II eplets mismatches (assessed by the HLA matchmaker algorithm) was a significant predictive factor for DSA formation (adjusted hazard ratio = 1.01, 95% confidence interval [1.00- 1.01], *P* < 0.0001). One hundred eighty patients (16.8%) received all the scheduled tests. In this subgroup, the detection of dnDSA was not different from others during the follow-up (13/180 [7.2%] vs. 64/892 [7.2%], *P* = 0.99).

**Table 1:** Main characteristics of patients with and without *de novo* donor specific antibodies

Variables	No dnDSA (*n* = 995)	dnDSA (*n* = 77)	Univariate analysis *P*-value
Recipient age, mean (±SD), yr	62.2 (14.3)	55.6 (17.5)	0.0018
Recipient gender, male (%)	693 (69.6)	62 (80.5)	0.166
Cause of end-stage kidney disease, *n* (%)			0.4278
- Diabetes	108 (10.9)	9 (11.7)	
- Vascular disease	136 (13.7)	9 (11.7)	
- Tubulo-interstitial disease	127 (12.8)	15 (19.5)	
- Glomerular disease	287 (28.8)	23 (29.9)	
- Hereditary kidney disease	225 (22.6)	11 (14.3)	
- Other	112 (11.3)	10 (12.9)	
Deceased donor, yes (%)	776 (78)	58 (75.3)	0.2735
Donor age, mean (±SD), yrs	55.6 ± 16.1	47.6 ± 18.5	0.0004
HLA mismatches, mean (±SD)	5.0 ± 1.7	5.7 ± 1.5	0.001
class I (A, B)	2.9 ± 1.0	3.2 ± 0.9	0.01
class II (DRB, DQB)	2.2 ± 1.2	2.5 ± 1.1	0.01
Molecular mismatches eplets load, mean (±SD)^a^			
Class I (A, B)	14.0 ± 6.9	16.0 ± 6.1	<0.0001
Class II (DRB, DQB)	6.0 ± 6.0	19.0 ± 11.0	<0.0001
Renal replacement therapy before transplantation, yes (%)	852 (85.6)	60 (77.9)	0.1045
Induction, yes (%)	573 (57.6)	58 (75.3)	0.3351
Polyclonal depleting antibodies, *n* (%)	89 (8.9)	9 (11.7)	0.4533
Basiliximab, *n* (%)	484 (48.6)	49 (63.6)	0.7662
Immunosuppressive therapy at discharge,			
Calcineurin inhibitors, *n* (%)	971 (97.6)	75 (97.4)	0.4504
- Tacrolimus	884 (88.8)	65 (84.4)	
- Cyclosporine	87 (8.7)	10 (13.0)	
Mycophenolic acid, *n* (%)	927 (93.2)	73 (94.8)	0.4482
mTOR inhibitors, *n* (%)	75 (7.5)	5 (6.5)	0.4489
Steroids, *n* (%)	987 (99.2)	77 (100)	0.4494
Immunosuppressive therapy at 1 year^a^			
Calcineurin inhibitors, *n* (%)	870 (92.0)	66 (89.1)	0.5095
- tacrolimus	839 (88.8)	64 (86.5)	
- cyclosporine	31 (3.3)	2 (2.7)	
Mycophenolic acid, *n* (%)	765 (81.0)	64 (86.5)	0.4535
mTOR inhibitors, *n* (%)	198 (21.0)	15 (20.3)	0.4491
Belatacept, *n* (%)	36 (3.8)	1 (1.4)	0.4477
Steroids, *n* (%)	921 (97.5)	74 (100)	0.4416
Immunosuppressive therapy at last follow-up			
Calcineurin inhibitors, *n* (%)	801 (80.5)	41 (53.2)	0.4958
- tacrolimus	792 (79.6)	41 (51.9)	
- cyclosporine	16 (1.6)	1 (1.3)	
Mycophenolic acid, *n* (%)	655 (65.8)	34 (44.2)	0.5196
mTOR inhibitors, *n* (%)	186 (18.7)	10 (13.0)	0.4409
Belatacept, *n* (%)	50 (5)	4 (5.2)	0.4503
Azathioprine, *n* (%)	13 (1.3)	0 (0)	0.4489
Steroids, *n* (%)	824 (82.8)	42 (54.5)	0.4942

dnDSA, *de novo* donor specific antibodies; HLA, human leukocytes antigen; mTOR, mammalian target of rapamycin.

HLA Eplets mismatches were assessed after imputation from low resolution to high resolution with the HLAr package using the HLA matchmaker algorithm (ABC Eplets matching v4.0, and DRDQDP Eplets matching v 3.1, www.epitopes.net).

a Data are expressed for the 1019 patients (945 without and 74 with dnDSA) with a functioning graft at 1year.

The median time from transplantation to dnDSA detection was 17 (IQR: 11–37) months. The rate of detection of dnDSA among all performed tests varied from 3.2% at month 12 to 0% between year 10 to 13 years (Figure 1b). The rate of dnDSA detection was similar regardless of the kit used, except at year 2 (2.6% with OneLambda, 0% with Immucor, *P* = 0.02, Supplementary Figure S2).

Next, we compared the incidence of DSA according to the test indication. Among the 4611 tests analyzed, 4267 were performed on a systematic basis (92.5%). dnDSA was detected in 46 of 4267 screening tests systematically realized (1.08%). It varied from 0% to 2.20% (Figure 1b). The maximal rate was observed at 1-year posttransplant. The epitope load was not different in class I or II according to the indication: the epitope load was 17.0 ± 6.2 (for cause) vs. 15.6 ± 6.1 (systematic screening), in class I, *P* = 0.47; and 20.4 ± 12.7 (for cause) vs. 18.8 ± 9.8 (systematic screening), *P* = 0.67 in class II. To note, we observed a trend of a higher class I and II epitope load for patients with class I and II DSA comparing with others (41.5 ± 10.5, 39.8 ± 11.8, and 33.3 ± 12.2 in the class I and II, class I, and class II groups, respectively; *P* = 0.053).

Among the 77 patients who developed dnDSA, immunizing factors were detected in 73 patients (94.8%) as follows: 28 patients had required blood or platelet transfusion before dnDSA detection, features of nonadherence to treatment were noted in medical records in 18 patients, and immunosuppression was minimized in 27 patients (calcineurin inhibitor withdrawal [*n* = 3] or low [<3 ng/ml] targeted doses [*n* = 24] for cancer or infection). Conversely, the occurrence of immunizing events was dramatically less common among the 995 patients who did not develop dnDSA (detected in 647 patients [65.0%], including 587 patients who required blood or platelet transfusion during the follow-up, 56 patients who required a minimization of immunosuppression, pregnancy [*n* = 3], and reported nonobservance [*n* = 1], *P* < 0.0001).

### Clinical and Histological Presentation According to the Mode of DSA Detection

The time between transplantation and dnDSA detection was similar between patients with dnDSA detected systematically or after testing for cause (Table 2). Mean fluorescence intensity of dnDSA was significantly higher in patients with dnDSA detected during a systematic screening (Table 2). In addition, kidney function was significantly better in patients in whom dnDSA was detected during the systematic screening (Table 2).

**Table 2:** Comparison between patients with *de novo* DSA detected during systematic or clinically indicated screening

Variables	Systematic screening (*n* = 46)	Clinically indicated (*n* = 31)	*P*-value
Recipient age, yr, mean (±SD)	57.2 ± 15.8	53.5 ± 19.8	0.35
Recipient gender, male, *n* (%)	40 (87)	22 (71)	0.14
Immunosuppressive therapy at dnDSA detection			
- Calcineurin inhibitors, yes (%)	38 (82.6)	26 (83.9)	0.99
- Mycophenolic acid, yes (%)	39 (84.8)	25 (80.6)	0.76
- mTORi, yes (%)	12 (26.1)	5 (16.1)	0.40
- steroids, yes (%)	46 (100)	31 (100)	>0.99
Time between transplantation and dnDSA detection, mo, median (IQR)	24(12–39)	12(6–36)	0.09
Serum creatinine at dnDSA detection (μmol/l), median (IQR)	135 (117–160)	255 (169–371)	<0.0001
Urine protein-to-creatinine ratio (mg/g)	174 (5–700)	385 (144–530)	0.11
dnDSA type, *n* (%)			0.12
class I	8 (17.4)	4 (12.9)	
class II	33 (71.7)	19 (61.3)	
class I and II	5 (10.9)	8 (25.8)	
Number of dnDSA, median (IQR)	1 (1–2)	1 (1–1)	0.33
MFI strength of immunodominant dnDSA, median (IQR)	8000 (4000–12,000)	2500 (1800–6500)	0.0006
MFI sum of dnDSA, median (IQR)	8000 (4000–12,000)	4000 (2000–8000)	0.02
Allograft biopsy after dnDSA detection,	39 (84.8)	27 (87.1)	0.99
yes (%)			
No specific changes	17 (43.6)	7 (25.9)	0.20
Borderline changes -Suspicion of TCMR	5 (12.8)	5 (18.5)	0.73
c4D positive without evidence of rejection	0	1 (2.6)	0.99
TCMR			0.71
grade I	3 (7.7)	3 (11.1)	
grade II	1 (2.6)	1 (3.7)	
AMR			0.80
Active	7 (17.9)	5 (18.5)	
Chronic active	5 (12.8)	2 (7.4)	
Chronic inactive	1 (2.6)	0	
Mixed	0	3 (11.1)	

ah, arteriolar hyalinosis; AMR, antibody-mediated rejection; cg, glomerular basement membrane double contours; ci, interstitial fibrosis; ct, tubular atrophy; cv, vascular fibrous intimal thickening; dnDSA, *de novo* donor specific antibodies; g, glomerulitis; HLA, human leukocytes antigen; i, interstitial inflammation; IQR, interquartile range; MFI, mean fluorescent intensity; mm, mesangial matrix expansion; mTORi, mammalian target of rapamycin inhibitors; ptc, peritubular capillaritis; t, tubulitis; TCMR, T cell-mediated rejection; ti, total inflammation; v, intimal arteritis.

**Figure 2:**
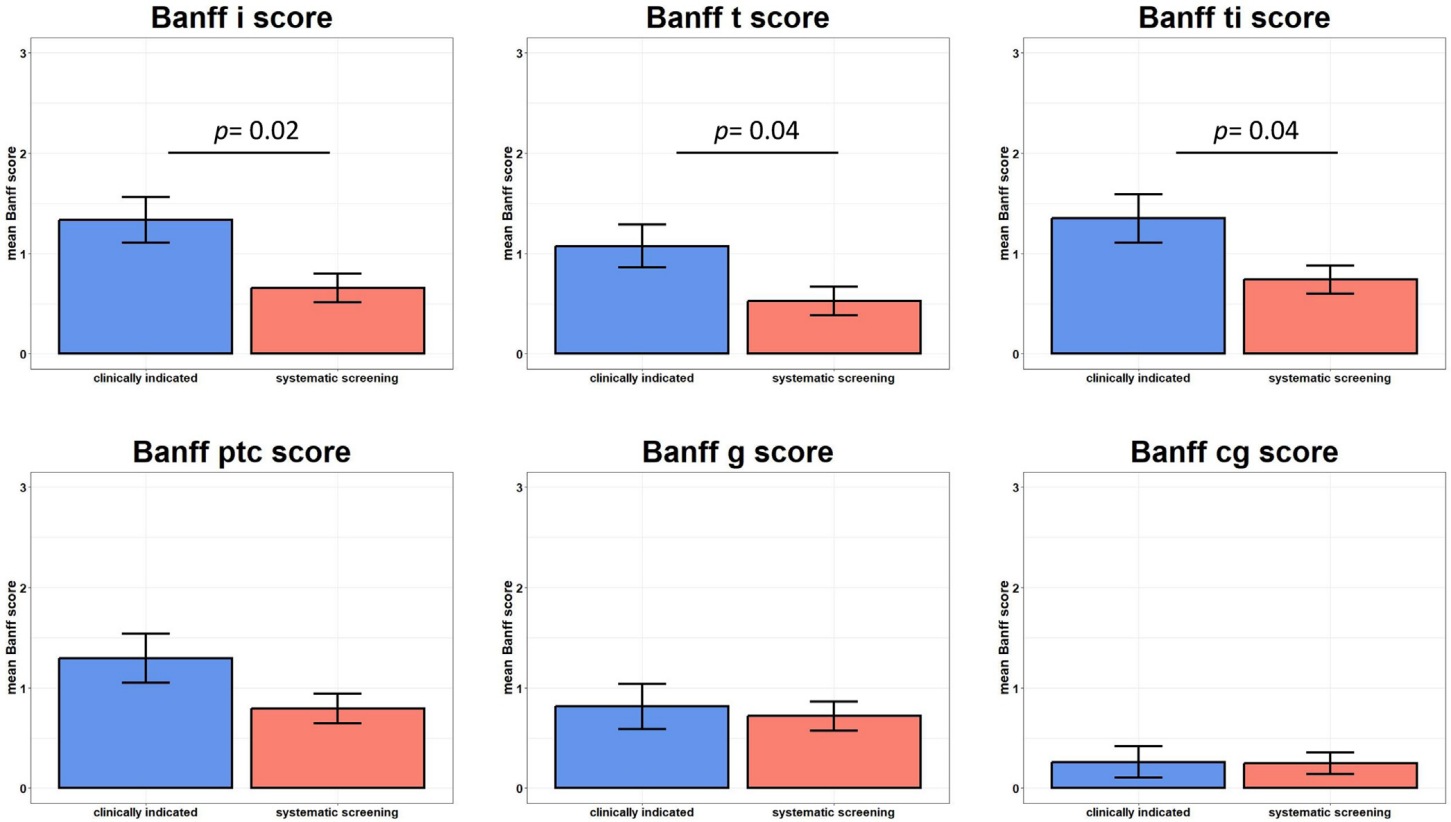
Main Banff scores at diagnosis biopsy grouped by test indication. Results are expressed in mean ± SD.

A kidney biopsy was performed in a large majority of patients after dnDSA detection (Table 2). As presented in Figure 2 and Supplementary Table S2, inflammation and tubulitis Banff scores were significantly higher in patients in whom dnDSA was tested for cause (i = 0.66 ± 0.88 vs. 1.33 ± 1.20, *P* = 0.02; t = 0.53 ± 0.89 vs. 1.10 ± 1.10, *P* = 0.04, respectively, in systematic vs. clinically indicated groups). However, glomerulitis and peritubular capillaritis were frequently observed in all patients with dnDSA, regardless of the reason for testing (g = 0.72 ± 0.92 vs. 0.81 ± 1.18, *P* = 0.72; ptc = 0.79 ± 0.92 vs. 1.30 ± 1.30, *P* = 0.09, respectively, in systematic vs. clinically indicated groups). Moreover, chronic glomerular changes (double contours) were similarly observed in both groups after systematic or clinically indicated testing, respectively. Biopsies without signs of rejection were observed in 17 of the 39 biopsies (43.6%) performed after a systematic detection of DSA and 7 of the 29 biopsies (25.9%) performed after detection of DSA tested for cause (*P* = 0.20). Similar results were obtained using the semisupervised clustering approach RejectClass tool, showing no significant difference in activity and chronic indexes between both groups (Supplementary Figure S3). A second biopsy was performed in 41 patients: 26 from the systematic screening DSA detection group, 6.7 (IQR: 2.7–12.4) months after the first biopsy. Twenty-one patients (51%) presented evidence of AMR: 12, 6, and 3 patients presented with lesions compatible with an active AMR, a chronic active AMR, and a chronic inactive AMR, respectively. There was no difference between the initial DSA detection modality and the presence of AMR in last follow-up biopsy: 15 of 26 of patients with dnDSA systematically detected and 6 of 15 of patients with dnDSA detected after testing for cause (*P* = 0.34).

After dnDSA detection, immunosuppression was increased in 33 of the 46 patients (71.7%) with dnDSA systematically detected and 25 of the 31 patients (80.6%) with dnDSA detected after testing for cause (Table 3). The increase of immunosuppression was mainly based on steroid pulse, plasma exchange, and B-cell depleting agents.

**Table 3:** Treatment after *de novo* DSA detection

Variables	Systematical screening (*n* = 46)	Clinically indicated (*n* = 31)	*P*-value
i.v. steroid pulses	20 (43.5)	20 (64.5)	0.07
T cell depleting polyclonal antibodies	1 (2.2)	5 (16.1)	0.03
B or plasma cell targeting Agent			0.49
Rituximab	27 (58.7)	21 (67.7)	
Bortezomib	1 (2.2)	0	
Intravenous Immunoglobulins	14 (30.4)	8 (25.8)	0.80
Plasma exchanges	23 (50.0)	17 (54.8)	0.65
Complement blockers	0	1 (3.2)	0.40
Tacrolimus introduction	11 (23.9)	7 (22.6)	>0.99
Disappearance of dnDSAs during the follow-up^a^, yes	24 (53.3)^a^	11 (35.5)	0.19
Time between detection and disappearance of dnDSAs, mo (IQR)	10.7 (8.1–15.3)	10.5 (5.2–19.9)	0.93

dnDSA, *de novo* donor specific antibodies; IQR, interquartile range.

Steroid pulses were administered at 10 mg/kg/d for 3 days, 2 mg/kg/d for 1 day, and followed by 1 week at 1 mg/kg/d ‘then tapered to 5 mg/d.

Rituximab was administered at 375 mg/m^2^ for 2 pulses at 1 week apart.

i.v. immunoglobulins were administered as a cure of 2 g/kg.

a Data are available for only 39 from the systematical screening group.

A follow-up of DSA was available for all except 1 patient. dnDSA was not detected during follow-up in 35 out of 77 patients (45.4%): 24 of 45 patients with dnDSA detected during systematic screening and 11 of 31 patients with dnDSA detected after testing for cause. dnDSA became undetectable more often among patients who had an intensification of immunosuppression (31/58 patients vs. 4/18 patients, *P* = 0.04).

**Figure 3:**
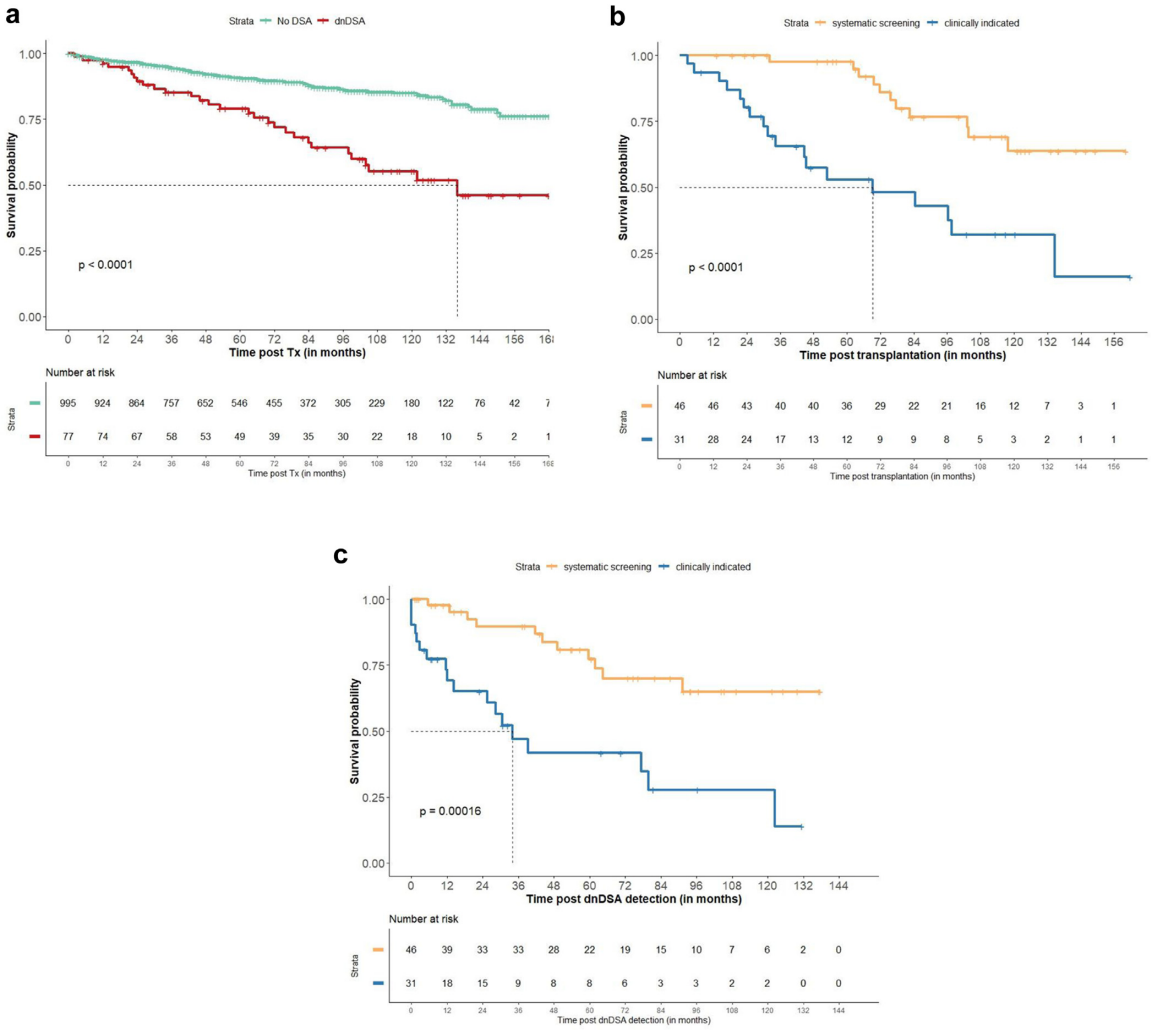
Death-censored kidney graft survival (a) from transplantation, and (b) from dnDSA detection, to the last follow-up, according to the type of detection. dnDSA, *de novo* donor specific antibodies.

We investigated the graft survival according to the dnDSA mode of detection. As expected, the death-censored graft survival was lower in patients who presented dnDSA (Figure 3a). However, both the death-censored kidney graft survival from transplantation (Figure 3b) and from the detection of dnDSA (Figure 3c) to the last follow-up were lower in patients who received a dnDSA detection for cause in comparison with others.

## Discussion

Whereas pure T cell-mediated rejection disappears with time posttransplantation, the occurrence of AMR continues,^23^, ^24^, ^25^ and is currently considered the major cause of graft loss.^2^ Preformed and *de novo* anti-HLA antibodies are the main components for the development of AMR.^8^ dnDSA can occur throughout the posttransplant period with a previously reported prevalence ranging from 11% to 20% depending on the duration of study period posttransplantation.^6^^,^^26^ Nonadherence to immunosuppressive therapy (and the related socioeconomic factors that contribute to nonadherence), donor-recipient HLA mismatches, blood transfusion, and previous T cell-mediated rejection episodes are considered to be predictive factors for dnDSA occurrence after transplantation.^7^^,^^27^^,^^28^ In the present study, the annual incidence (from 0% to 3.2%) and prevalence (7.1%) of dnDSA is lower than in previous reports. This can be explained by the high proportion of patients, given the standard triple immunosuppressive regimen, including calcineurin inhibitors, the development of educational programs, and avoiding immunosuppressive minimization strategies. As a result, the observed incidence of dnDSA detected by systematic protocol screening in our transplant population is very low (46 tests positive for 4267 [1.08%] in tests performed during a follow-up period of 8 (5–11) years, meaning that 100 systematic tests are required to detect 1 DSA. The rate of positive tests in case of annual systematic detection was even lower after 1 year posttransplantation. In addition, in most patients with dnDSA detected after systematic screening, an immunizing event or features of nonadherence were observed before dnDSA detection. Moreover, patients who developed dnDSA during the follow-up presented with a higher A/B and DR/DQB1 epitope load. The epitope load was not different regarding the test indication. This is probably explained by the absence of consideration of the epitope load for DSA screening during this retrospective study. Recently, Wiebe and colleagues investigated in a retrospective single-center cohort of 1029 recipients the role of HLA-DR/DQ molecular mismatch and age to predict the risk for dnDSA formation. They defined different recipient-age mismatch categories and found that patients with high class II epitope load (≥15 HLA DQA/B eplets mismatches, any HLA DRB1/3/4/5) presented with the highest risk for dnDSA formation compared with low risk (<8 HLA DQA/B eplets mismatches and <6 HLA DRB1/3/4/5 mismatches, hazard ratio = 6.36, 95% confidence interval [3.7–10.8], *P* < 0.00001), or intermediate risk (≤14 HLA DQA/B eplets mismatches, any HLA DRB1/3/4/5, hazard ratio = 2.56, 95% confidence interval [1.6–4.2], *P* < 0.0002).^22^ This suggests that the epitope load approach could be an interesting tool for selecting recipients that could really benefit from a systematic dnDSA screening. In the recently published OuTSMART study,^29^ Stringer and colleagues found a similarly low incidence of DSAs (5.8%). Nonetheless, our data suggest an interest in the systematic screening for some patients. Indeed, despite a low number of patients, we observed a better death-censored graft survival in patients (from both the transplantation and the dnDSA detection) in patients with dnDSA receiving a systematic screening in comparison with others. Nevertheless, we cannot exclude some biases in this retrospective study that could explain in part this difference (e.g., the detection of transitional or low or nonpathologic dnDSA, a better kidney function at diagnosis, in the systematic group). Moreover, due to the low number of patients for whom dnDSA were detected from a systematic screening, we were not able to address the impact of the proposed treatment in those patients. Taken together and considering the high cost of Luminex-based anti-HLA antibody screening, our results suggest that, after 1 year, systematic screening should be proposed only in patients at high risk of developing dnDSA, that is, noncompliant patients, those having an immunizing event such as a blood transfusion, or those in whom immunosuppression is voluntarily reduced. Some other tools such as tacrolimus intrapatient variability could be useful to detect and improve patient adherence to treatment^30^ before dnDSA detection.

The aim of annual screening for anti-HLA antibodies is to detect dnDSA before the presence of AMR lesions or at very early stages of AMR.^13^^,^^31^ However, despite a better death-censored graft survival, our data do not perfectly support this idea, because we observed acute and chronic histological AMR features in patients that received a biopsy after dnDSA detection during systematic screening. Lesions of transplant glomerulopathy, illustrated by a cg score >0 were seen in up to 15% of biopsies in this group, similar to what was observed in the clinically indicated group. Chronic AMR remains a clinical challenge because no treatment has thus far demonstrated its effectiveness.^11^ These results are consistent with those recently reported by Stringer *et al.*^29^ In this last study, the authors assessed the role of intervention therapy after detection of dnDSA. Intervention therapy was based on steroid boost, increased maintenance immunosuppression, and an interview to explain the importance of medication adherence. However, despite these interventions, the development of dnDSA was still associated with progression to graft failure.^29^ Similar results from a single-center retrospective study were reported by Cun *et al.*^32^ In a cohort of 464 patients followed-up with for 5 (1–19) years using a low mean fluorescent intensity cut-off at 500, the prevalence of dnDSA was 11.9%. Only 44% of patients with dnDSA underwent a kidney biopsy and therapeutic intervention was done in only 3.9% of the cohort.

Our study presents several limitations. First, this is a retrospective single-center study. However, during this period we applied standard immunosuppression protocols that suggest generalization of our results. Second, we defined patients with a low immunological risk as those without preformed anti-HLA antibodies. However, we did not exclude patients who have previously received blood transfusion or women with a past of pregnancy. Thus, we cannot rule out that some recipients could have been considered as patients with a donor-specific cellular memory.^4^^,^^5^ However, to ensure the completeness of such information is a complex task in practice. The definition of low immunological risk patients remains a matter of debate. By using other recently described tools (e.g., PIRCH II scores, or Eplet load), we could probably have a slightly different study population. Nonetheless, none of these tools are fully validated for clinical practice. Third, all patients did not receive all the scheduled screening tests, especially after the first year posttransplantation. Thus, we cannot rule out that some patients could have developed dnDSA without patent renal consequences during follow-up. The low rate of dnDSA in patients with stable kidney function, who did not present sensitizing events, would explain the absence of adoption of this recommendation from all transplant physicians. Moreover, the lack of difference regarding the rate of dnDSA detection in patients who received all the scheduled tests and patients who did not; as well as the absence of clear difference regarding histological features of AMR, whatever the mode of detection (clinically indicated or not) of dnDSA detection, do not support the mass, unselected, screening strategy. In addition, the lack of efficient treatment of chronic AMR limits the utility of systematic screening. Regarding the low number of patients who developed dnDSA, we were not able to further compare the outcomes regarding its disappearance, particularly in case of AMR. Future studies addressing this point, as well as the utility of new injury markers, such as donor-derived cell free DNA, are urgently needed at least in the high-risk selected population.^33^^,^^34^ Moreover, the added-value of molecular phenotypes in case of normal or subnormal biopsies could help in the future to encourage clinicians to optimize immunosuppression, if necessary, in case of detection of dnDSA.^35^

In conclusion, our results suggest that the detection of dnDSA after yearly systematic screening in low immunological risk kidney transplant patients is a rare event. Systematic screening for dnDSA seems to have a limited impact to detect AMR at an earlier stage.

## Disclosure

All the authors declared no competing interests.

## Supplementary Materials

Supplementary File (PDF)

**Figure S1.** Study flowchart.

**Figure S2.** Rate of detection of *de novo* DSA with the OneLambda (A) or the Immucor (B) manufacturer during follow-up (in yr) for all patients**.**

**Figure S3.** Histological patterns defined by the semisupervised RejectClass tool of DSA positive biopsies grouped by the type of detection.

**Table S1.** Predictive factors for *de novo* DSA development.

**Table S2.** Mean Banff scores grouped by the type of detection of DSA.
