# Supplementary material for: Utility of Routine Post Kidney Transplant Anti-HLA Antibody Screening
Source: Kidney Int Rep. 2024 Feb 18;9(5):1343–53. doi: 10.1016/j.ekir.2024.02.1394 (PMC11068955; doi:10.1016/j.ekir.2024.02.1394)

Supplementary material

Supplementary Figure 1. Study flowchart.

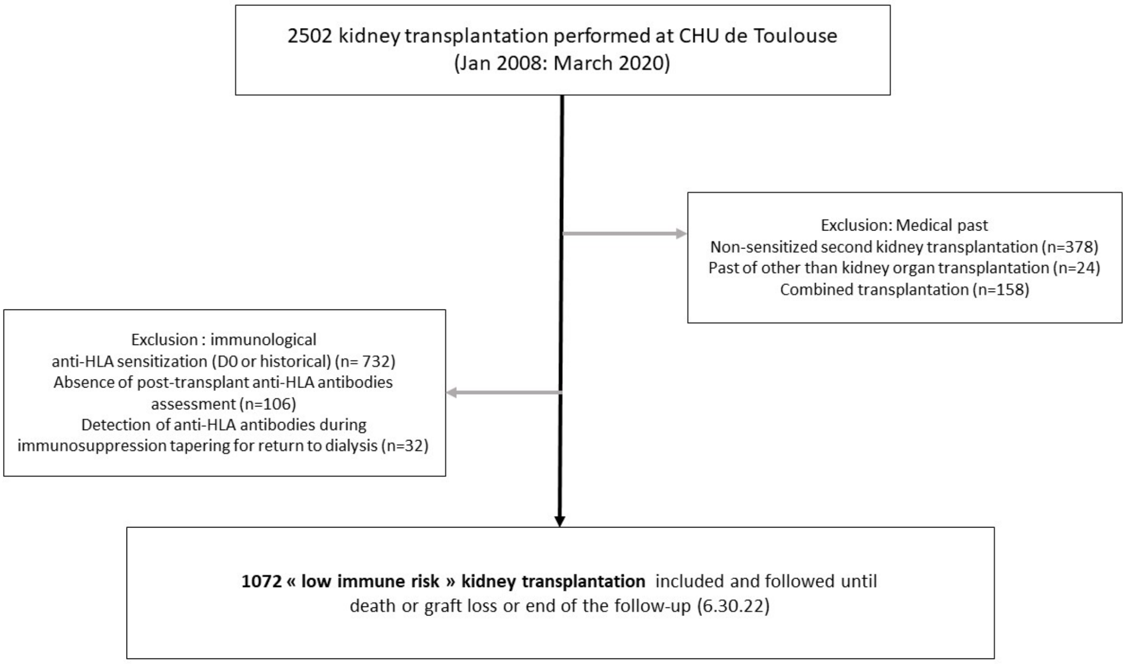

Supplementary Figure 2. Rate of detection of *de novo* DSA with the OneLambda (A) or the Immucor (B) manufacturer during follow-up (in year) for all patients. Results are similar in all timepoints except at year 2 (2.6% with OneLambda®, 0% with Immucor, p=0.02).

Results are expressed as the percentage of positive tests among all performed tests.

Abbreviation: dnDSA, *de novo* DSA

A)

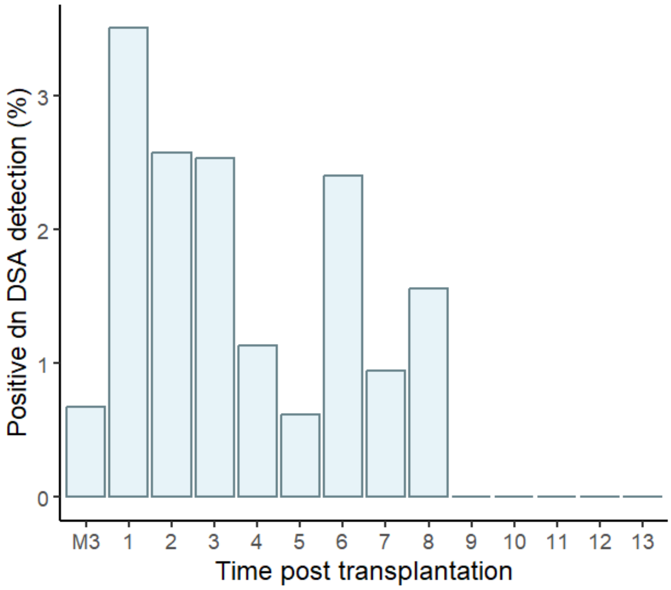

B)

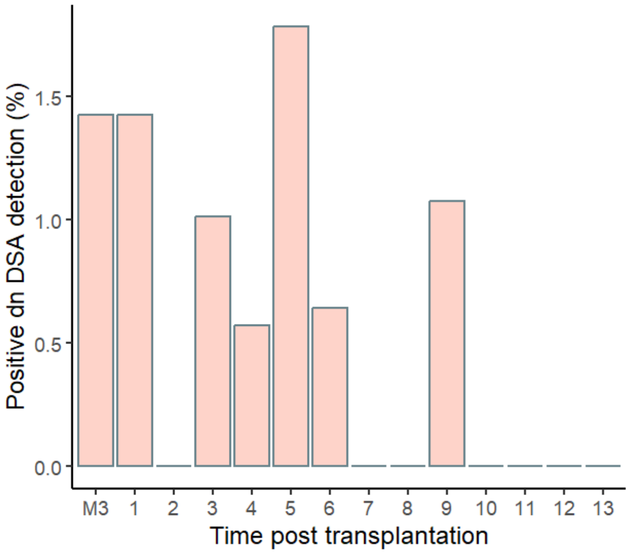

Positive dn DSA detection (%)
0.0
0.5
1.0
1.5
M3
1
2
3
4
5
6
7
8
9
10
11
12
13
Time post transplantation

Supplementary Table 1. Predictive factors for *de novo* DSA development.

The best-fit model according to the AIC score (877) included the sum of HLA mismatches eplets estimated by the HLA matchmaker algorithm, the recipient, and the donor age.

AIC scores of Models 1, 2, and 3 described in the patients and method section were 903, 905 and 902, respectively.

Proportional-hazards assumption of the model was graphically tested and presented below.

Abbreviations: Emm_S, Sum of HLA class I and II eplets mismatches; age D, Donor Age; age_R, Recipient age at transplantation

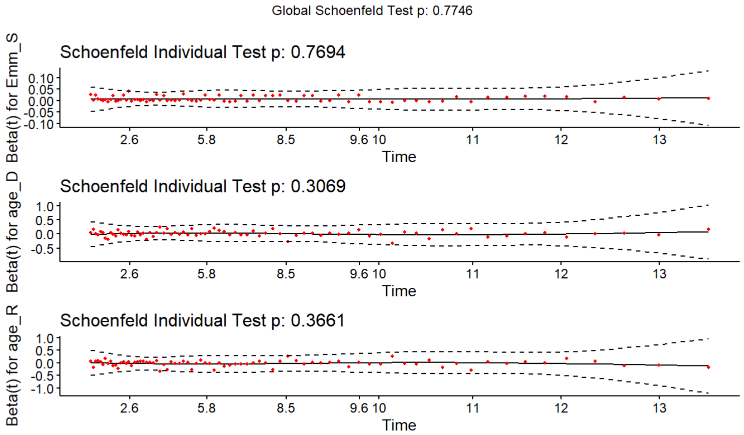

	aHR	*Lower.95 CI*	*Upper .95 CI*	*p*
HLA matchmaker Eplets mismatches	1.01	1.00	1.01	<0.0001
Recipient age	0.97	0.95	0.99	0.01
Donor age	1.01	0.95	0.99	0.31

Supplementary Table 2. Mean Banff scores grouped by the type of detection of DSA.

Variables	Systematic screening (n= 46)	Clinically indicated (n= 31)	*p*-value
Main Banff scores			
g mean (± SD)	0.72 ± 0.92	0.81 ± 1.18	0.72
i mean (± SD)	0.66 ± 0.88	1.33 ± 1.20	0.02
t, mean (± SD)	0.53 ± 0.89	1.10 ± 1.10	0.04
v mean (± SD)	0.09 ± 0.28	0.30 ± 0.70	0.13
ptc mean (± SD)	0.79 ± 0.92	1.30 ± 1.30	0.09
cg mean (± SD)	0.25 ± 0.65	0.25 ± 0.80	0.96
ci mean (± SD)	0.50 ± 0.61	0.50 ± 0.80	0.72
ct mean (± SD)	0.55 ± 0.61	0.65 ± 0.63	0.43
cv mean (± SD)	0.28 ± 0.52	0.54 ± 0.90	0.25
ah mean (± SD)	1.38 ± 1.23	0.69 ± 1.01	0.02
mm mean (± SD)	0.42 ± 0.73	0.61 ± 1.00	0.78
ti, mean (± SD)	0.74 ± 0.77	1.30 ± 1.10	0.04
positive C4d, yes (%)	19 (41.3)	11 (40.7)	0.62
C4d score, mean (± SD)	0.94 ± 1.12	0.80 ± 1.19	0.62

Supplementary Figure 3. Histological patterns defined by the semi-supervised RejectClass tool of DSA positive biopsies grouped by the type of detection (A, C: Acute and Chronic components in biopsies performed for DSA detect from systematic screening; B, D, Acute and Chronic components in biopsies performed for DSA detected from clinically indicated reason.). Each biopsy is projected in the form of a point on the respective graph.

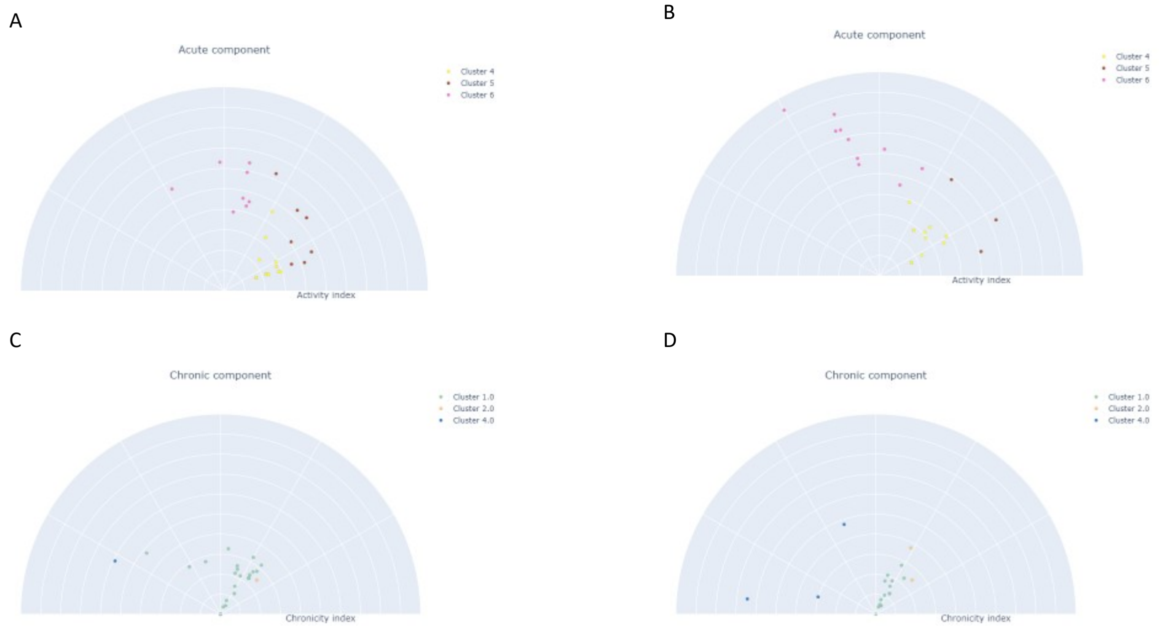

Supplement: Supplementary File (PDF) [file mmc1.pdf]
